# Controlled phase evolution from Cu_0.33_Co_0.67_S_2_ to Cu_3_Co_6_S_8_ hexagonal nanosheets as oxygen evolution reaction catalysts†

**DOI:** 10.1039/c9ra00640k

**Published:** 2019-03-27

**Authors:** Jingjing Feng, Yu Meng, Zixuan Lian, Liang Fang, Ziyao Long, Yongtao Li, Yun Song

**Affiliations:** Department of Materials Science, Fudan University Shanghai 200433 China; School of Materials Science and Engineering, Anhui University of Technology Maanshan 243032 China; Shanghai Innovation Institute for Materials Shanghai 200444 P. R. China

## Abstract

Developing cheap and efficient transition metal-based catalysts for the oxygen evolution reaction (OER) plays the key role in large-scale implementation of hydrogen production. However, there is still a lack of effective ways to tune the catalysts performance for the OER reaction from the aspect of structure design and element modulation simultaneously. Herein, a novel Cu_0.33_Co_0.67_S_2_ hexagonal nanosheet has been synthesized through the coprecipitation reaction followed by subsequent vapor sulfidation. Simply mixed with carbon nanotubes (CNTs) during electrode preparation, this Cu_0.33_Co_0.67_S_2_ exhibits an overpotential of 284 mV *vs.* RHE at a current density of 10 mA cm^−2^ in 1.0 M KOH. The improved OER performance of the Cu_0.33_Co_0.67_S_2_ electrode can be attributed to the electrocatalytically active sites involved in octahedral coordination structures and further activated by Cu substitution. The encouraging results provide insight into further rational design of transition metal-based electrochemical catalysts towards OER applications.

## Abstract 2

Developing cheap and efficient transition metal-based catalysts for the oxygen evolution reaction (OER) plays the key role in large-scale implementation of hydrogen production.

## Graphical abstract

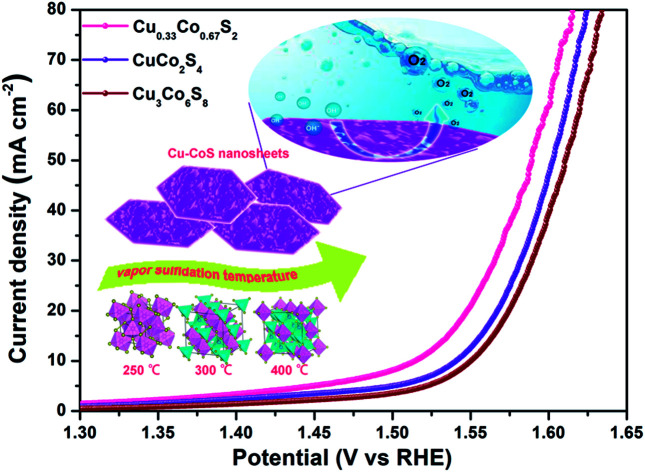

## Introduction

The depletion of conventional fossil fuels has sparked extensive research for renewable and clean energies, such as hydrogen energy, which has high gravimetric energy density.^1,2^ Electrochemical water splitting has been considered as a promising avenue to directly produce hydrogen from abundant seawater. To gain efficient water splitting, great efforts have been devoted to exploring low-cost and high performance electrocatalysts for the hydrogen evolution reaction (HER) and oxygen evolution reaction (OER), in which OER is the bottleneck for the kinetically sluggish four-proton-coupled electron transfer in alkaline medium.^3–8^ Meanwhile, relatively high activation energy is needed to generate the O=O double bond.^9,10^

Among various OER catalysts, cobalt based sulfides have been widely investigated for the high catalytic activity and low cost.^11–15^ Moreover, the structure diversity of cobalt based sulfides offers more option for improving OER performance. With the aid of electrodeposition, Co–S with amorphous structure and nanosheet morphology was synthesized, exhibiting an overpotential of 361 mV at 10 mA cm^−2^ for the OER in 1.0 M KOH.^16^ Liu *et al.* synthesized the pure Co_9_S_8_ phase with the overpotential of 278 mV at 10 mA cm^−2^.^17^ Wang *et al.* employed Co_3_S_4_ as OER catalysts with an overpotential of 430 mV,^18^ while the CoS_2_ with overpotential of 278 mV is tested at the similar testing condition.^19^ Here comes a question that is there any relationship between structure and OER catalytical performance of cobalt based sulfides? Han *et al.* have pointed out that the precondition for this comparison is to synthesize various cobalt based sulfides with similar morphology.^20^ Given the multiple forms of cobalt sulfides, a big challenge still exists in the field of precisely controlling phase and morphology. For instance, Golberg *et al.* found Co_9_S_8_ is the product of incomplete sulfurization using Na_2_S as the sulfur source, while CoS_2_ is the product of over sulfurization using thioacetamide as the sulfur source.^21^ Nevertheless, it is rather difficult to obtain the single phase of cobalt based sulfides by using the same sulfur source. To solve this problem, Han *et al.* demonstrated that single phase of Co_9_S_8_, Co_3_S_4_ and CoS_2_ can be simply prepared through one-step facile hydrothermal method by just changing the amount of carbon disulfide (CS_2_) to 0.13 ml, 0.3 ml and 0.8 ml, respectively.^20^ However, CS_2_ is highly toxic, which even minor leak leads to danger. Convenient and safety method is still needed to controllable synthesize cobalt sulfides with desired phase and morphology.

Recently, the research hotspot has been transferred from single cobalt-based sulfides to binary metal sulfide, as introducing another transition metal in cobalt-based sulfides is an effective approach to further enhance the catalytic activity for OER.^22–24^ Amongst, the advantage of Cu element can be expressed as: low-cost, non-toxic and the similarity in atomic size and electro-negativity between Cu and Co.^25^ Peng *et al.* have proved that the addition of Cu can supply extra catalytic sites in Cu–CoS nanosheets, eventually leading to high performance in sensitized solar cells.^26^ It has been proved by theoretical calculation that intrinsic metallic nature and more band states near Fermi level could be obtained by introducing Cu to ensure the fast charge transfer, which will play an important role in charge transfer in OER performance.^27,28^ Moreover, Zhang *et al.* have demonstrated that the introduction of Cu element into CoS_2_ can enhance the activity of Co-sites and simultaneously activate the inert S-sites in CoS_2_. Introducing Cu into CoS_2_ is demonstrated to optimize the active species adsorption free energy of CoS_2_ catalyst.^28^ To the best of our knowledge, few investigations have been focused on the OER performance and the involved catalytic mechanism of Cu–Co–S system. The critical reason is that the controllable synthesis of single cobalt sulfides is still a big challenge, not to mention the bimetallic Cu–Co–S system which containing some complicated reaction between two metals. For instance, Bezverkhyy *et al.* indicated that the instead of Cu_3_Co_6_S_8_ pure phase which is previously desired, mixture of Cu_2_S and Co_9_S_8_ eventually formed upon sulfidation process of the Cu_0.33_Co_0.67_S_2_ precursor.^29^ Thus, it is rather desired but still a challenge to synthesize Cu–Co–S with a similar morphology and a controlled pure phase, which can be used to tune the catalysts performance for OER reaction from the aspect of structure design and element modulation.

Herein, Cu-substituted cobalt sulfide hexagonal nanosheets with three different structures crystallographic (Cu_0.33_Co_0.67_S_2_, CuCo_2_S_4_ and Cu_3_Co_6_S_8_) have been successfully synthesized and their OER catalytical performance have been compared. Amongst, Cu_0.33_Co_0.67_S_2_ exhibits the lowest overpotential of 284 mV *vs.* RHE at a current density of 10 mA cm^−2^ in 1.0 M KOH, as compared with that of 310 and 320 mV in CuCo_2_S_4_ and Cu_3_Co_6_S_8_ phase, respectively. The improved OER performance of Cu_0.33_Co_0.67_S_2_ electrode can be attributed to the electrocatalytically active sites involved in octahedral coordination structure and further activated by Cu substitution. These encouraging results shed light on further rational design of transition metal-based electrochemical catalysts towards OER application.

## Experimental

### Synthesis of Cu_0.33_Co_0.67_(OH)_2_ precursor

Firstly, the Cu_0.33_Co_0.67_(OH)_2_ precursor was prepared by a facile coprecipitation process according to the previous report.^30^ Specifically, 5 mmol CoCl_2_·6H_2_O (Aladdin, 99.99% metals basis), 2.5 mmol CuCl_2_·2H_2_O (Aladdin, 99.99% metals basis), and 45 mmol hexamethylenetetramine (HMT) (Aladdin, ≥99.0%) were dissolved in the oxygen-removal deionized water. This prepared solution was refluxed with nitrogen at 120 °C for 5 h under continuous magnetic stirring in a three-necked flask, and then cooled down to room temperature naturally. After filtrating, the coprecipitation product was centrifugally washed with deionized water and alcohol for several times and then dried at 60 °C overnight to obtain the Cu_0.33_Co_0.67_(OH)_2_ precursor powder.

### Synthesis of copper cobalt sulfides

The series of copper cobalt sulfides were synthesized by thermal annealing of Cu_0.33_Co_0.67_(OH)_2_ precursor powders with sulfur powders (Aladdin, 99.95% metals basis). For the preparation of Cu_0.33_Co_67_S_2_, 50 mg Cu_0.33_Co_0.67_(OH)_2_ precursor powders and 100 mg sulfur powders were separately put on opposite ends of a quartz boat in CVD furnace, which was keeping at 250 °C for 3 h with the heating rate of 2 °C min^−1^ under a H_2_/Ar atmosphere, and then cooled down naturally to obtain the black power of Cu_0.33_Co_67_S_2_. Similarly, CuCo_2_S_4_ and Cu_3_Co_6_S_8_ can be synthetized at the temperature of 300 °C, 350 °C, 400 °C and 500 °C in the same way, respectively.

### Material characterization

The phase structures of the obtained samples were characterized by X-ray diffraction (XRD, Bruker D8 Advance X-ray diffractometer) with Cu Kα radiation (*λ* = 1.5406 Å, 0.02° per step) and further refined by a Rietveld program RIETAN-2000.^31^ To examine their morphologies and microstructures, scanning electron microscopy (SEM, FEI Nova Nano Sem 450) and transmission electron microscopy (TEM, FEI Tecnai G2 F 20 S-Twin) were carried out, respectively. And the elemental composition and chemical states of the samples were measured by X-ray photoelectron spectroscopy (XPS, PHI 5000C EACA system) with a C 1s peak at 284.6 eV as the standard signal.

### Preparation of working electrodes

To improve the conductivities of the as-prepared samples, carbon nanotubes (CNTs) (XFNANO, >95%, length 10–30 μm) were added before preparing the catalyst slurry, as reported previously.^32^ In detail, 6 mg sample of Cu_0.33_Co_0.67_S_2_ sheet and 1 mg CNTs were dispersed in 4 ml absolute ethanol, with ultrasonic dispersion for 4 h to form a uniform mixture, and then dried at 60 °C. Fig. S1† shows the Cu_0.33_Co_0.67_S_2_ sheet is bonded tightly with conductive CNTs, indicating that the 2D morphology sample can be easily coupled with conductive carbon nanotubes upon preparation. To noted that the following electrochemical measurements of as-prepared catalysts in this work were processed by mixing with CNTs.

The catalyst slurry was prepared by a 30 minutes ultrasonic process of the mixture of 4 mg catalyst, 970 μL anhydrous ethanol and 30 μL Nafion solution Nafion (Sigma-Aldrich, ∼5 wt%) under 40 °C. Subsequently, 8 μL as-prepared catalyst slurry was coated uniformly on the glassy carbon electrode with the area of 7 mm^2^ and dried at room temperature, and the working electrode was totally loading about 0.03 mg active materials.

### Electrochemical measurements

The electrochemical performances of as-obtained catalysts were conducted on an electrochemical workstation (CHI760e) with a three-electrode cell configuration in oxygen-saturated 1 M KOH (Aladdin, GR 95%) solution at 25 °C, in which the slurry-coated glassy carbon electrode, a graphite rod and a saturated calomel electrode (SCE) were respectively used as working electrode, counter electrode and reference electrode. Linear sweep voltammetry (LSV) measurement was performed at a scan rate of 10 mV s^−1^. Subsequently, the obtained OER LSV data were treated with iR-compensation according to the equation: *E*_c_ = *E*_m_ − *I*_m_ × *R*_s_, where *E*_c_, *E*_m_, *I*_m_ and *R*_s_ stand for the compensated voltage, measured voltage, measured current and electrolyte resistance, respectively. Unless specified otherwise, all the reported potentials were calibrated with reference to the reversible hydrogen electrode (RHE) based on the following equation: *E*_RHE_ = *E*_SCE_ + 0.059 × pH + *E*_m_. And the turnover frequencies (TOF) values were calculated according to the following equation: TOF (s^−1^) = (*j* × *A*)/(4 × *F* × *n*), where *j*, *A*, *F* and *n* represent the current density at a given overpotential, the geometric surface area of the working electrode, *F* is the Faraday constant, mole number of transition metal(s) loaded on the electrode, respectively. Moreover, cyclic voltammograms (CVs) of samples were carried out at different scanning rates (2, 4, 6, 8 10 and 20 mV s^−1^), and the apparent electrochemical double layer capacitance (*C*_dl_) was estimated at non-faradaic region. Half of the difference between charging current density and discharging current density (|*j*_c_ − *j*_d_|/2) was plotted linearly *versus* scan rates, and *C*_dl_ value was reflected from the fitted slope. The electrochemical impedance spectra (EIS) were obtained in a frequency range from 10 mHz to 100 kHz at the amplitude of the sinusoidal voltage of 5 mV. The durability of Cu_0.33_Co_0.67_S_2_ was performed in current over 8 h at a fixed potential at 310 mV in 1 M KOH.

## Results and discussion

### Structural and morphological characterizations upon phase transformation

**Fig. 1:**
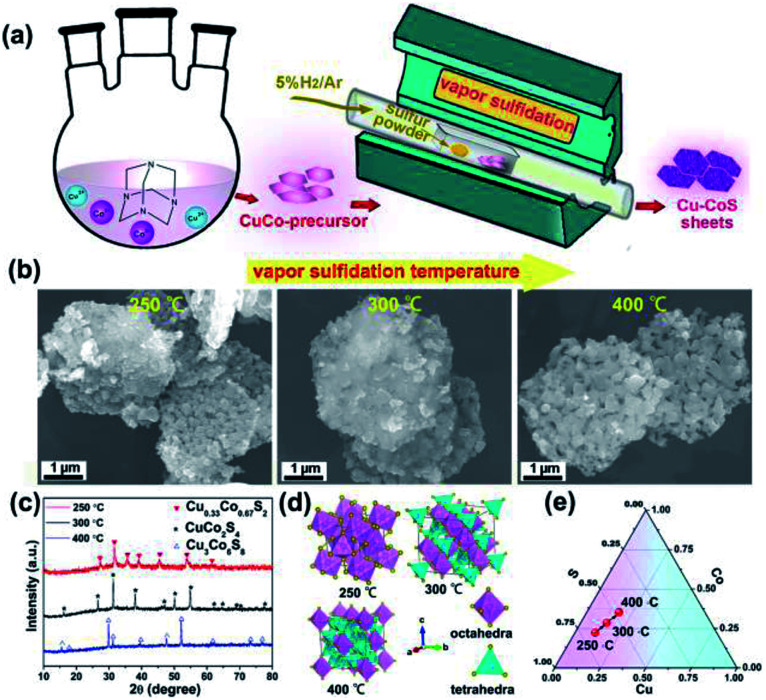
(a) Schematic illustration of the preparation; (b) morphology evolution upon thermal annealing; (c) XRD patterns of Cu_0.33_Co_0.67_S_2_, CuCo_2_S_4_ and Cu_3_Co_6_S_8_, respectively; (d) schematic unit cell of Cu_0.33_Co_0.67_S_2_, CuCo_2_S_4_ and Cu_3_Co_6_S_8_ phase, respectively; (e) compositional design map adopted in Cu–Co–S.

The copper cobalt sulfides with different crystal structures were prepared by simple coprecipitation reaction followed by subsequent thermal annealing with sulfur powders, as illustrated in Fig. 1a. Firstly, CuCo-precursor was prepared by coprecipitation method with hexamethylenetetramine as alkaline source. The crystal structure and morphology of this CuCo-precursor were examined, as shown in Fig. S2† Obtained from XRD pattern, the CuCo-precursor can be assigned to a brucite Cu_0.33_Co_0.67_(OH)_2_ structure, consistent with previous reported works.^33^ Moreover, the morphology of the Cu_0.33_Co_0.67_(OH)_2_ exhibits two-dimensional hexagonal sheet structure. Then, the Cu_0.33_Co_0.67_(OH)_2_ hexagonal sheets, mingled with sulfur at fixed ratio of 1 : 2, are subjected to thermal annealing, at the temperature ranged from 250 °C to 400 °C. The morphology evolution upon thermal annealing at different temperature is evaluated in Fig. 1b, in which the two-dimensional hexagonal sheet morphology is still maintained without significant difference between pristine Cu_0.33_Co_0.67_(OH)_2_ precursor. The crystal structure upon thermal annealing was characterized by XRD, as shown in Fig. 1c. Initially, under the low temperature of 250 °C, the diffraction peaks can be assigned to the standard JCPDS no. 89-1492, indicating a cubic pyrite phase with the *Pa*3̄ space group. It should be noted here that this phase is identical to CoS_2_ phase, how to ensure the introduction of Cu? The uniform distribution of Co, Cu and S elements were further confirmed by energy-dispersive X-ray (EDX) spectra, as illustrated in Fig. S3.† The schematic crystal structure of Cu_0.33_Co_0.67_S_2_ (250 °C) is described in Fig. 1d, in which Metal atoms (Cu/Co) locate at the center site, surrounded by six sulfur atoms in the octahedral arrangement, which are connected with each other through sharing corners.^34^ Lifted the annealing temperature to 300 °C, all diffraction peaks can be indexed to the standard JCPDS no. 42-1450 (CuCo_2_S_4_), indicating a normal spinel phase with the *Fd*3*m* space group. The schematic crystal structure is that Cu atoms occupy the tetrahedral sites while the Co atoms still locate at the octahedral sites.^34^ Further increasing the annealing temperature to 400 °C, all diffraction peaks can be assigned to the standard JCPDS no. 02-1459, which crystallized in the cubic *Fm*3̄*m* structure. The Cu_3_Co_6_S_8_ consists of tetrahedron and octahedron, in which every 24 tetrahedrons share the 6 corners of each octahedron.^34,35^ Based on above phase evolution information, the compositional design map adopted in Cu–Co–S can be depicted in Fig. 1e. On the premise of fixed mass ratio of initial reactant (CuCo-precursor and sulfur powder), with annealing temperature raised, controlled phase evolution from Cu_0.33_Co_0.67_S_2_ to Cu_3_Co_6_S_8_, with CuCo_2_S_4_ as intermediated phase can be realized in our vapor transformation method. It is observed that under such lower sulfide annealing temperature (lower than 400 °C), the Cu is expected to substitute the Co position, as confirmed by the combination of previous results of XRD pattern and EDX mapping. Increasing the annealing temperature to 500 °C, the phase segregation occurs with the appearance of new Cu_1.96_S phase, as shown in Fig. S4.†

**Fig. 2:**
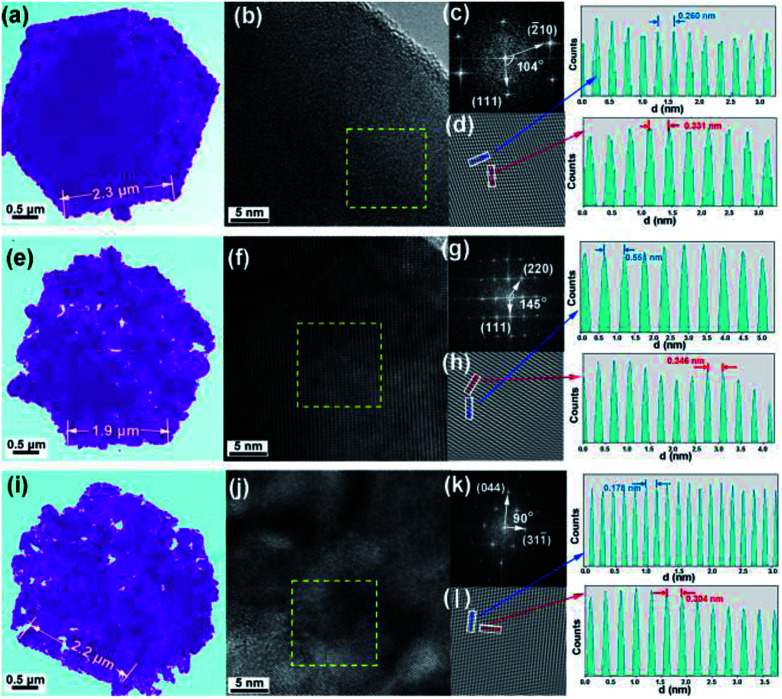
(a) Typical TEM image, (b) high-resolution TEM image, (c) fast Fourier transform (FFT) and (d) their lattice images transferred by inverse fast Fourier transform (IFFT) of Cu_0.33_Co_0.67_S_2_; (e) typical TEM image, (f) high-resolution TEM image, (g) fast Fourier transform (FFT) and (h) their lattice images transferred by inverse fast Fourier transform (IFFT) of Cu CuCo_2_S_4_; (i) typical TEM image, (j) high-resolution TEM image, (k) fast Fourier transform (FFT) and (l) their lattice images transferred by inverse fast Fourier transform (IFFT) of Cu_3_Co_6_S_8_.

To further detect the morphology and structure evolution upon vapor transformation, the high-resolution transmission electron microscopy is employed, as shown in Fig. 2. Firstly, the Cu_0.33_Co_0.67_S_2_ shows regular hexagonal morphology, as shown in Fig. 2a. The side length of this hexagon is estimated to be 2 μm, agrees with previous SEM observation. Fig. 2b manifests microstructure of Cu_0.33_Co_0.67_S_2_ with conspicuous lattice spacing, which shows the crossed lattice fringes of 0.260 nm and 0.331 nm, assigned to the (−210) and (111) interplanar *d*-spacings, as illustrated in Fig. 2c and d. It should be noted here that the value of these observed *d*-spacings is slightly larger than that of the standard CoS_2_ lattice (JCPDS no. 89-1492). Moreover, the CuCo_2_S_4_ still exhibits hexagonal morphology, as shown in Fig. 2e, indicating the thermal and structural stability upon annealing. Fig. 2f depicts the microstructure of Cu_0.33_Co_0.67_S_2_ showing the crossed lattice fringes of 0.346 nm and 0.551 nm, indexed to the (220) and (111) interplanar *d*-spacings, as illustrated in Fig. 2g and h. Finally, the end product of Cu_3_Co_6_S_8_ with maintained hexagonal morphology is observed in Fig. 2i. Fig. 2j depicts the microstructure of Cu_3_Co_6_S_8_ showing the crossed lattice fringes of 0.178 nm and 0.304 nm, indexed to the (044) and (311) interplanar *d*-spacings, as illustrated in Fig. 2k and m. The selected area electron diffraction (SAED) patterns of the Cu_0.33_Co_0.67_S_2_, CuCo_2_S_4_ and Cu_3_Co_6_S_8_, as shown in Fig. S5,† agree well with above XRD analysis, further confirming the feasibility of our controlled phase evolution method.

**Fig. 3:**
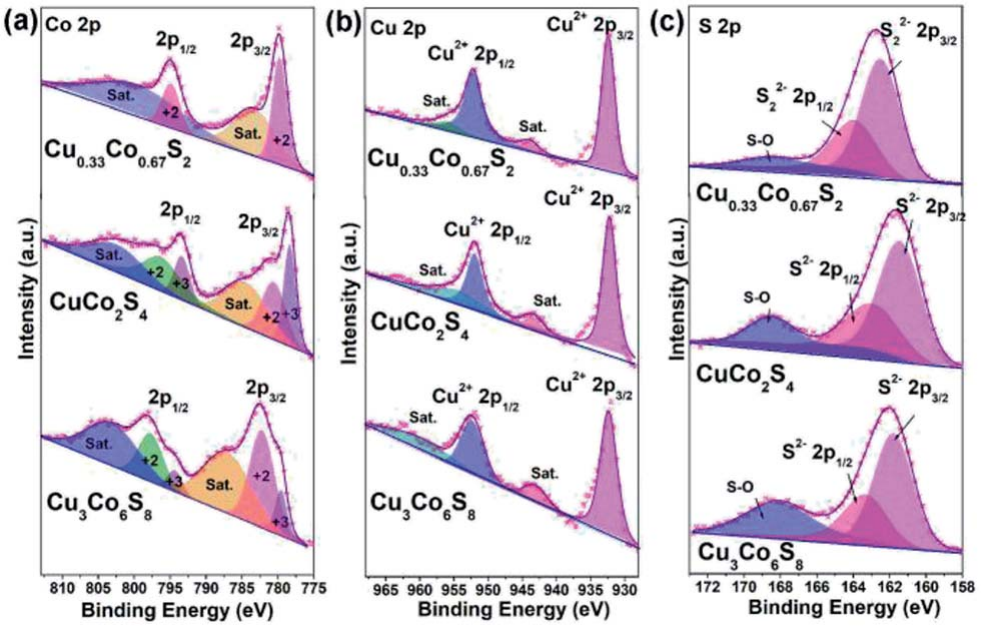
High-resolution XPS spectrum of (a) Co 2p; (b) Cu 2p and (c) S 2p of as-obtained Cu–Co–S.

To detect the elemental compositions and the chemical states of Cu–Co–S sheets, X-ray photoelectron spectroscopy (XPS) is carry out. As obtained in Fig. 3a, the high-resolution Co 2p spectrum for Cu_0.33_Co_0.67_S_2_ exhibits two peaks located at 779.8 eV and 794.9 eV, which belong to the Co^2+^ 2p_3/2_ and Co^2+^ 2p_1/2_ states.^36^ On the other hand, the fitting results Co 2p spectra reveal that the Co^3+^ and Co^2+^ cations coexist both in CuCo_2_S_4_ and Cu_3_Co_6_S_8_, respectively.^37^ Regarding the Cu 2p spectra in Fig. 3b, Cu^2+^ exists in Cu_0.33_Co_0.67_S_2_, CuCo_2_S_4_ and Cu_3_Co_6_S_8_.^38^ And in Fig. 3c, the high-resolution spectra of S 2p for Cu_0.33_Co_0.67_S_2_ reveals the existence of S_2_^2−^ species, while the S 2p spectra of CuCo_2_S_4_ and Cu_3_Co_6_S_8_ confirms the existence of S^2−^ species instead of S_2_^2−^ species.^37^ In addition, the peak at about 168.5 eV belongs to the peak of S–O bonding, which might owing to the exposure to air.^39^

### The effect of structure modification on OER performance

**Fig. 4:**
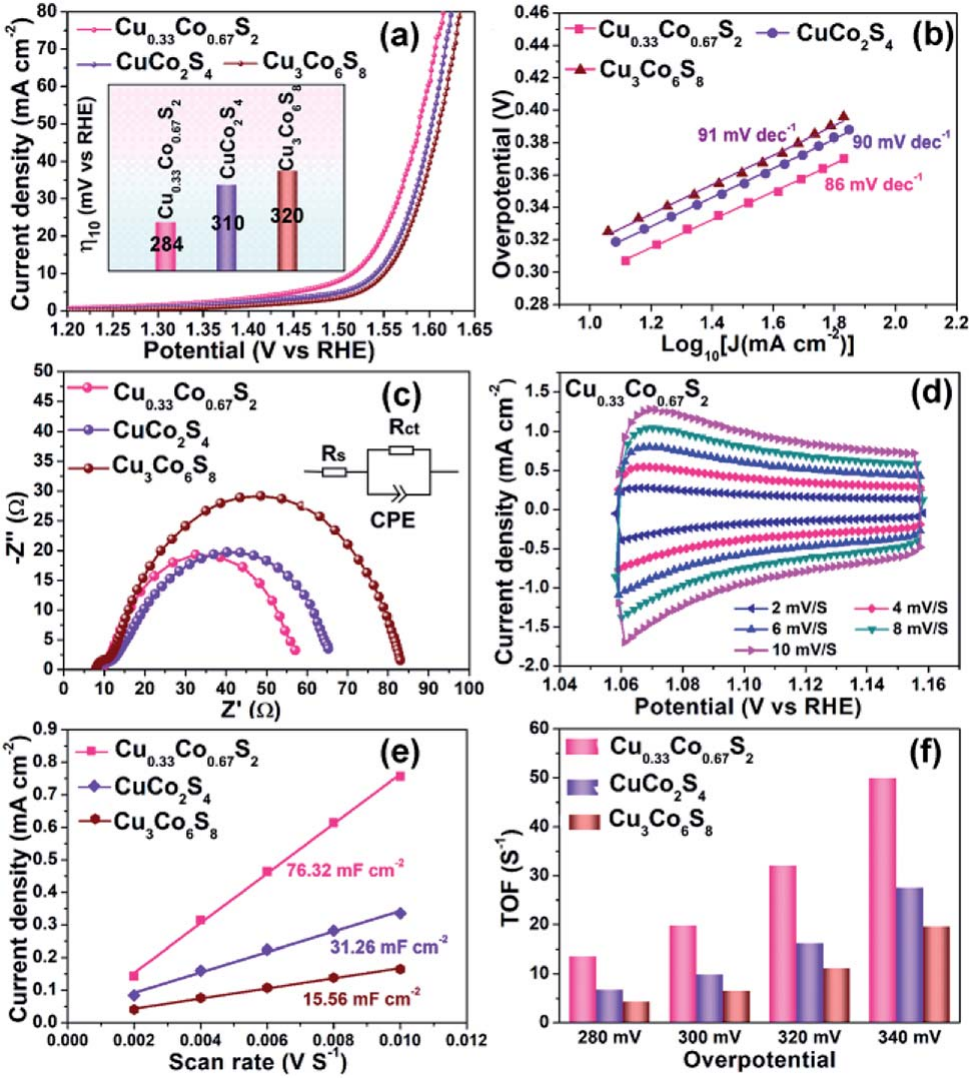
OER performance comparison of Cu_0.33_Co_0.67_S_2_, CuCo_2_S_4_ and Cu_3_Co_6_S_8_: (a) LSV curve, inserted with overpotential@10 mA cm^−2^; (b) Tafel analysis; (c) Nyquist curves of EIS; (d) CVs of Cu_0.33_Co_0.67_S_2_ at various scanning rates; (e) *C*_dl_ value comparison; (f) TOF values evaluated at various overpotential.

To investigate the relationship between crystal structure and oxygen evolution reaction (OER) activity, the as-prepared Cu_0.33_Co_0.67_S_2_, CuCo_2_S_4_ and Cu_3_Co_6_S_8_ samples were evaluated in a standard three-electrode system with 1 M KOH electrolyte, respectively. Fig. 4a presents the linear sweep voltammetry (LSV) curves, in which the Cu_0.33_Co_0.67_S_2_ manifests the optimal catalytic activity, with an overpotential of 284 mV at 10 mA cm^−2^. This overpotential value is lower than 310 mV and 320 mV of CuCo_2_S_4_ and Cu_3_Co_6_S_8_ samples, respectively. Moreover, the Tafel slope, which is an indicative value of catalytic kinetics of OER,^40^ of Cu_0.33_Co_0.67_S_2_ CuCo_2_S_4_ and Cu_3_Co_6_S_8_ phases are compared in Fig. 4b, where a smaller Tafel slope represents the greatly increased OER rate. The lower Tafel slope value for Cu_0.33_Co_0.67_S_2_ (86 mV dec^−1^) indicates that the kinetics is more favorable as compared to that of CuCo_2_S_4_ (90 mV dec^−1^) and Cu_3_Co_6_S_8_ (91 mV dec^−1^), respectively. This enhanced OER rate performance of Cu_0.33_Co_0.67_S_2_ is attributed to the smaller charge transfer between the active sites, as evidenced in smaller loop in EIS observation (Fig. 4c). To further quantify the kinetics, the equivalent circuit model is employed with calculated solution resistance (*R*_s_) and a charge transfer resistance (*R*_ct_), as shown in Table S1.† The sequence of the *R*_ct_ value obtained from the diameter of the semicircles in the high frequency zone is Cu_0.33_Co_0.67_S_2_ (47 Ω) < CuCo_2_S_4_ (54 Ω) < Cu_3_Co_6_S_8_ (72 Ω), demonstrating faster electron transfer process upon OER for Cu_0.33_Co_0.67_S_2_.

The effective electrochemical active site is the key for the OER performance.^41,42^ Thus, to further unravel the possible causation of the differences in OER catalytic performance in term of the crystal structure, the electrochemical surface area (ECSA) and turnover frequencies (TOF) have been evaluated, respectively. To estimate the ECSA, the cyclic voltammograms measurements of these products are carried out by sweeping the potential across the non-faradaic region in static solution. Fig. 4d shows the CVs of Cu_0.33_Co_0.67_S_2_ and Fig. S6† shows CVs of CuCo_2_S_4_ and Cu_3_Co_6_S_8_, respectively, which revealed that the Cu_0.33_Co_0.67_S_2_ has a faster increase in current density at different scan rates. On the other hand, the capacitive behavior and OER performance of the as-synthesized three Cu–Co–S samples are compared according to CVs of Cu–Co–S product in Fig. S7.† Compared with CuCo_2_S_4_ and Cu_3_Co_6_S_8_, the Cu_0.33_C_o0.67_S_2_ exhibits the well-defined rectangular shape, implying that a series of active sites with different levels of energies are formed at lower potentials.^43^ And the *C*_dl_ was calculated from cyclic voltammetry (CV) curves *versus* different scan rates, which can be used to estimate the value of ECSA.^44^Fig. 4e shows the values of *C*_dl_ which obtained from the value of the slop of linear plots, with a tendency of as Cu_3_Co_6_S_8_ (15.56 mF cm^−2^) < CuCo_2_S_4_ (31.26 mF cm^−2^) < Cu_0.33_Co_0.67_S_2_ (76.32 mF cm^−2^), indicating the more active catalytic sites were obtained in Cu_0.33_Co_0.67_S_2_ for OER.

Fig. 4f compares the TOF values estimated at various overpotentials, which manifests the intrinsic activities of catalytical sites. It is obvious that the TOF value of Cu_0.33_Co_0.67_S_2_ is higher than CuCo_2_S_4_ and Cu_3_Co_6_S_8_ samples at different testing overpotential. Combining ECSA and TOF value of three samples, it is demonstrated that remarkably improved active catalytic sites are located in Cu_0.33_Co_0.67_S_2_, responsible for the enhanced OER performance with respect to lower overpotential and Tafel slope. Obtained from above results, some information can be provided: firstly, Cu_0.33_Co_0.67_S_2_, CuCo_2_S_4_ and Cu_3_Co_6_S_8_ samples all show acceptable OER catalytical performance with overpotential ranged from 284 mV to 320 mV at 10 mA cm^−2^, which are ahead of the most of as-reported transition metal compounds, as listed in Table S2.† This can be attributed to their 2D hexagonal sheet morphology in some degree. Their 2D flaky morphology can increase the accessibility of the active sites owing to small diffusion barrier to the substrate molecules, further providing a high active site density.^45^ Secondly, given the same 2D morphology of the three samples, the performance difference of the three samples is originated from the crystal structural differentiation. In Cu_0.33_Co_0.67_S_2_ structure cell, the metal ions are all located in the MS_6_ (M = Cu/Co) octahedra, while for CuCo_2_S_4_, two-third metal ions are located at the centre of the MS_6_ (M = Cu/Co) octahedra. The lowest ratio of MS_6_ (M = Cu/Co) octahedra of one-ninth is obtained in Cu_3_Co_6_S_8_. Thus, relationship between crystal structure and oxygen evolution reaction (OER) activity can be established as: the octahedrally coordinated metal sites show higher activity when compared with that of tetrahedrally coordinated metal sites. Specifically, the more octahedral coordination structure MS_6_ is, the better the catalytic effect can be obtained.^46^

To further verify the tendency mentioned above, a new sample is synthesized at the temperature of 350 °C for 3 h in the same way as synthetizing Cu_0.33_Co_0.67_S_2_. The XRD pattern (Fig. S8a†) and further Rietveld refinement (Fig. S8b†) indicate that this new counterpart sample contains two phases which are CuCo_2_S_4_ and Cu_3_Co_6_S_8_, respectively, and the account of them are 37.31 wt% and 62.69 wt%. Thus, it can be calculated that the proportion of MS_6_ in the product is about 44.76%. Subsequently, the LSV measurements of the product at 350 °C is conducted under the same condition. The product at 350 °C exhibits the catalytic activity with an overpotential of 318 mV at 10 mA cm^−2^ (in Fig. S8c†). And the relation between the OER performance (overpotential) and the ratio of octahedral coordination structure is illustrated in Fig. S8d,† showing that with the ratio of MS_6_ (M = Cu/Co) octahedra increasing from 11% to 100%, the overpotentials at 10 mA cm^−2^ decreases from 320 to 284 mV.

### Influence of copper introduction on OER performance

**Fig. 5:**
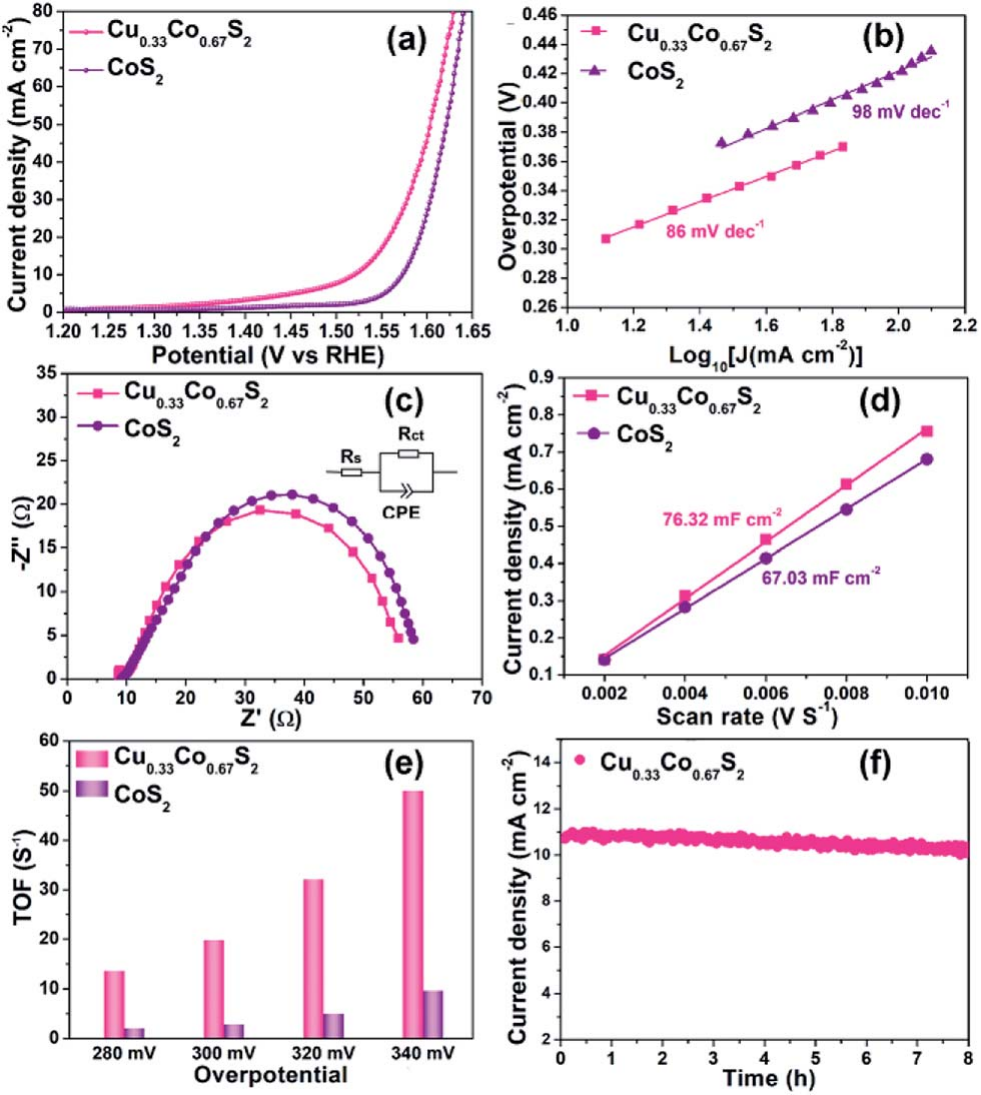
OER performance comparison of Cu_0.33_Co_0.67_S_2_ and CoS_2_: (a) LSV curve, inserted with overpotential@10 mA cm^−2^; (b) Tafel analysis; (c) Nyquist curves of EIS; (d) ECSA value comparison; (e) TOF values evaluated at various overpotential; (f) cyclic stability of Cu_0.33_Co_0.67_S_2_.

In spite of the MS_6_ (M = Cu/Co) octahedra provide more active sites, there is still an open question that what is the role played by the Cu introduction in OER performance. To further elucidate the indispensable effect of Cu elements, the referential pyrite CoS_2_ has been synthesized through the same procedure without the addition of Cu element. Cu_0.33_Co_0.67_S_2_ and CoS_2_ were prepared at the same sulfidation temperature of 250 °C to minimize the effect of geometric aspects. As compared in Fig. 5a, Cu_0.33_Co_0.67_S_2_ displays an overpotential of 284 mV at the current density of 10 mA cm^−2^, outperforming 343 mV of pure CoS_2_. The Tafel slopes are fitted to be 86 mV dec^−1^ and 98 mV dec^−1^ with and without Cu addition, as described in Fig. 5b. These OER performance comparisons indicate more favorable OER activity is obtained after addition of Cu, which is attributed to the smaller charge transfer between the active sites, as evidenced in smaller loop in EIS observation (Fig. 5c). This enhanced charge transfer was rationally explained by previously reported theoretical calculation that intrinsic metallic nature and more band states near Fermi level is obtained by introducing Cu to ensure the fast charge transfer.^47^ Moreover, Zhang *et al.* have demonstrated that the introduction of Cu element into CoS_2_ can enhance the activity of Co-sites and simultaneously activate the inert S-sites in CoS_2_.^28^

Based on above results, it can be concluded that structure design and element modulation both are effective ways to tune the catalytical performance for OER. Amongst, structure plays the prerequisite role in determining the number of catalytical sites, including practical and potential catalytical sites. In our Cu–Co–S system, the Cu_0.33_Co_0.67_S_2_ with all the metal ions located in the MS_6_ (M = Cu/Co) octahedra, shows the most fascinating OER performance. With the ratio of MS_6_ (M = Cu/Co) octahedra increasing from 11% to 100%, the overpotential at 10 mA cm^−2^ decreases from 320 to 284 mV. Thus, the octahedrally coordinated metal sites show higher activity as compared with that of tetrahedrally coordinated metal sites, which are evidenced in ESCA and TOF results. Subsequently, Cu_0.33_Co_0.67_S_2_ and CoS_2_, with the same structure that all the metal ions located in the MS_6_ (M = Cu/Co) octahedra, still show the performance difference. This difference is attributed to the effect of Cu substitution, activating more catalytical sites which are previously inert. The number of catalytical sites is basically determined by MS_6_ (M = Cu/Co) octahedra. This can be observed in the ESCA value which represents the practical and potential catalytical sites show slightly increased after Cu substitution, however, dramatical increment is observed in TOF value implying potential catalytical sites are triggered by Cu.

**Fig. 6:**
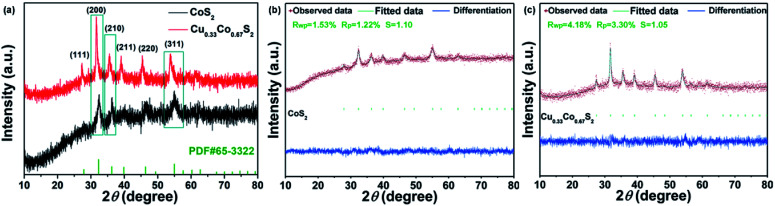
(a) XRD patterns of Cu_0.33_Co_0.67_S_2_ and CoS_2_; Rietveld refinement of the XRD pattern of (b) CoS_2_ and (c) Cu_0.33_Co_0.67_S_2_.

The electrochemical surface area (ECSA) and turnover frequencies (TOF) were compared for Cu_0.33_Co_0.67_S_2_ and CoS_2_. The cyclic voltammetry (CV) curves *versus* different scan rates for pure CoS_2_ are shown in Fig. S9.†Fig. 5d shows the *C*_dl_ values of 76.32 mF cm^−2^ for Cu_0.33_Co_0.67_S_2_, which is slightly higher than that of 67.03 mF cm^−2^ for CoS_2_. The addition of Cu indeed leads to more active catalytic sites in some degree, however, the increment is not as high as that of structure modulation, as shown in Fig. 4e. On the other hand, obtained from Fig. 5e, it can be clearly seen that TOF values of Cu_0.33_Co_0.67_S_2_ are much higher than CoS_2_ at different testing overpotentials. Thus, it is demonstrated that as compared with structure modulation, the addition of Cu actually boosts intrinsic activities of catalytical sites, as evidenced in TOF analysis. The durability of Cu_0.33_Co_0.67_S_2_ is shown in Fig. 5f, in which Cu_0.33_Co_0.67_S_2_ can retained for 90% of the initial value over 8 h, exhibiting long-term cycling stability for OER. There still exists a question why the addition of Cu increases the intrinsic activity of CoS_2_ as shown in TOF analysis. To elucidate the underlying mechanism of Cu addition, the XRD patterns of Cu_0.33_Co_0.67_S_2_ and CoS_2_ are compared in Fig. 6a. It can be seen that Cu successfully substitutes into the CoS_2_ without changing the pristine cubic phase, however, obvious peak shift to lower angles can be observed after Cu addition, indicating that the interlayer distances were increased. Further Rietveld refinement is employed to provide the detail crystal expansion after Cu substitution, as shown in Fig. 6a and c. Corresponding parameters obtained from Rietveld refinement are listed in Table S2.† The CoS_2_ is a cubic phase with lattice parameter of *a* = *b* = *c* = 5.610 Å, while Cu atoms substitute for Co atoms in CoS_2_ matrix to form Cu_0.33_Co_0.67_S_2_, with slightly expanded lattice parameters of *a* = *b* = *c* = 5.625 Å. This increment in lattice parameter is due to that the ionic radius of Cu is larger than that of Co, thus the lattice is expanded when the Cu atoms substitute for Co atoms in CoS_2_ matrix.^48^ The estimated lattice expansion ratio reaching 1% is obtained in Cu_0.33_Co_0.67_S_2_ as compared with that of CoS_2_, in which lattice distortion and lattice tensile strain are involved.^49^ More recently, Fan *et al.* have demonstrated that massive lattice distortion lead to activate more catalytical sites which were previously inert, eventually boosts electrochemical performance.^50^ Moreover, with respect to lattice tensile strain, it can facilitate active intermediates adsorption, such as O*, OH* and OOH*, by enhancing binding strength between functional catalyst to active intermediates.^51^

## Conclusion

In conclusion, we have successfully synthesized series of copper-substituted cobalt sulfide compounds, from Cu_0.33_Co_0.67_S_2_, CuCo_2_S_4_ to Cu_3_Co_6_S_8_ with the similar hexagonal sheet shape. Benefited from the similar morphology, the relationship between structure and OER catalytical performance has been established. The Cu_0.33_Co_0.67_S_2_ with all the metal ions located in the MS_6_ (M = Cu/Co) octahedra manifestes the optimal OER performance, with lowest overpotential of 284 mV at a current density of 10 mA cm^−2^, indicating the MS_6_ (M = Cu/Co) octahedra exhibits higher catalytic activity. Moreover, the effect of Cu substitution is elucidated to trigger previously inert sites in MS_6_ (M = Cu/Co) octahedra by introducing lattice distortion and lattice tensile strain. The comprehensive investigation on OER performance of Cu–Co–S provide insight into further rational design of transition metal-based electrochemical catalysts towards OER application.

## Conflicts of interest

There are no conflicts to declare.

## Supplementary Material

RA-009-C9RA00640K-s001
