# Supplementary material for: Controlled phase evolution from Cu0.33Co0.67S2 to Cu3Co6S8 hexagonal nanosheets as oxygen evolution reaction catalysts
Source: RSC Adv. 2019 Mar 27;9(17):9729–36. doi: 10.1039/c9ra00640k (PMC9062190; doi:10.1039/c9ra00640k)
Supplement: RA-009-C9RA00640K-s001 [file RA-009-C9RA00640K-s001.pdf]

Electronic Supplementary Material (ESI) for RSC Advances.
This journal is © The Royal Society of Chemistry 2019

Supporting Information

Controlled Phase Evolution from $Cu_{0.33}Co_{0.67}S_2$ to $Cu_3Co_6S_8$ Hexagonal Nanosheet as Oxygen Evolution Reaction Catalysts

Jingjing Feng,[1ab] Yu Meng,[1a] Zixuan Lian,[a] Liang Fang,[b] Ziyao Long,[a] Yongtao Li,[*b] Yun Song[*ac]

[a.] *Department of Materials Science, Fudan University, Shanghai 200433, China*

[b.] *School of Materials Science and Engineering, Anhui University of Technology, Maanshan 243032, China*

[c.] *Shanghai Innovation Institute for Materials, Shanghai 200444, P. R. China*

[1.] *These two authors have equal contribution to this work*

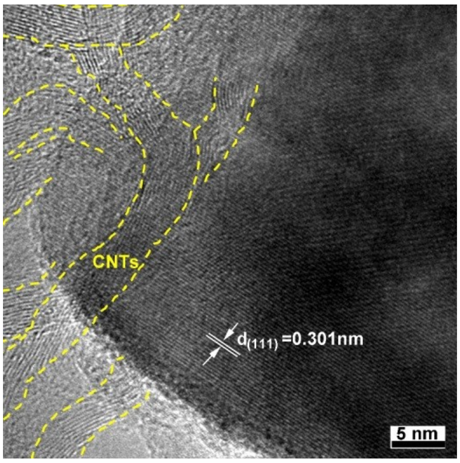

Fig. S1. high-resolution TEM image of $Cu_{0.33}Co_{0.67}S_2$/CNT.

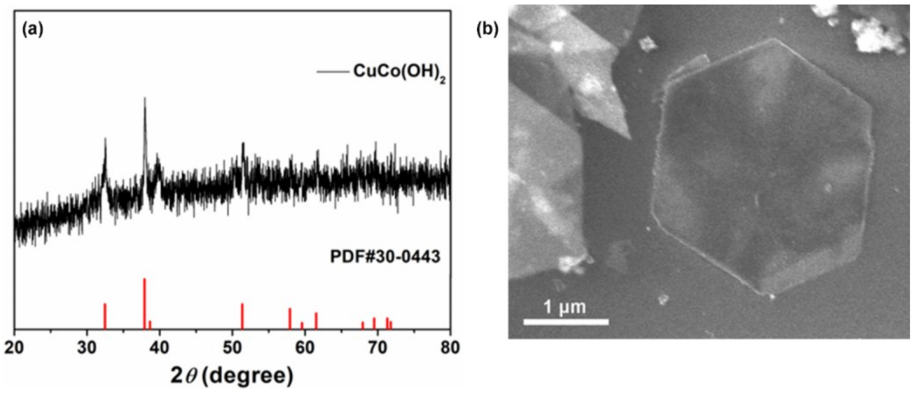

Fig. S2. (a) XRD pattern and SEM image of $CuCo(OH)_2$.

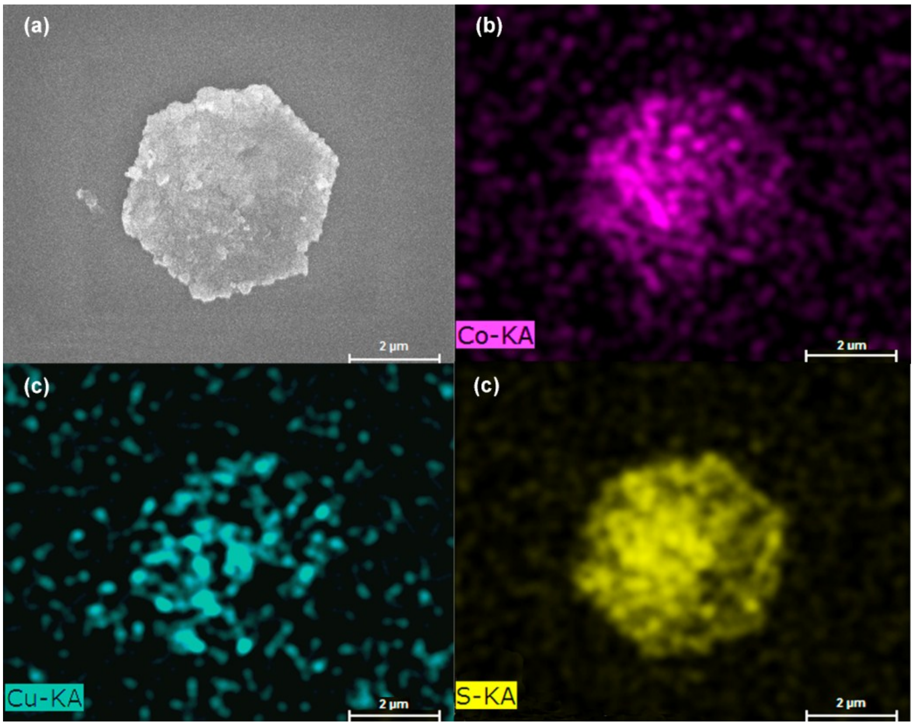

Fig.S3. (a)HAADF-SEM image and (b–d) elemental mapping images of $Cu_{0.33}Co_{0.67}S_2$.

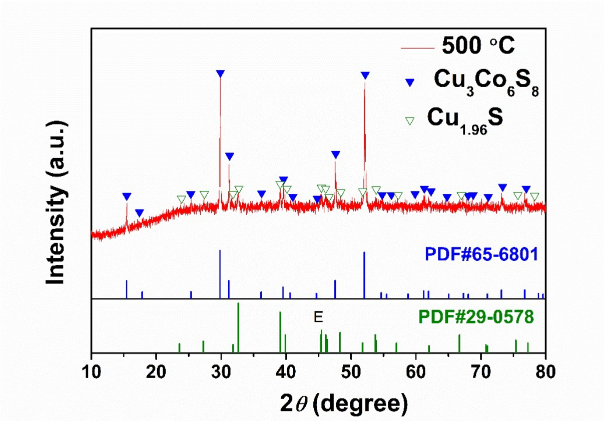

Fig. S4. XRD pattern of the Cu-Co-S product at 500 °C.

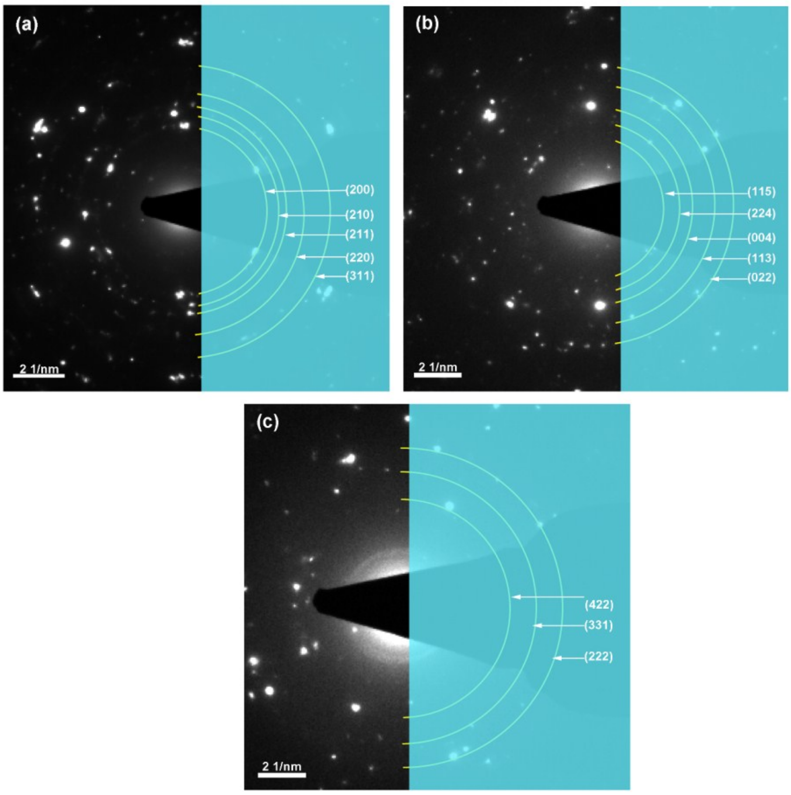

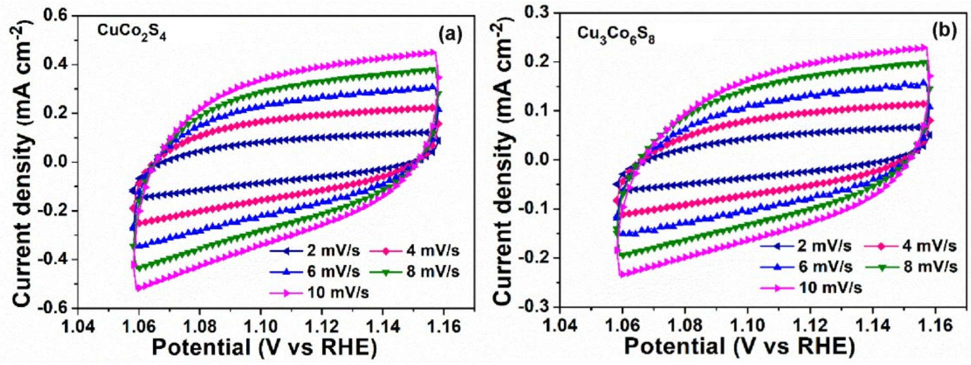

Fig. S5. the corresponding SAED pattern of (a) $Cu_{0.33}Co_{0.67}S_2$, (b) $CuCo_2S_4$ and (c) $Cu_3Co_6S_8$.

Fig. S6. CVs of (a) $CuCo_2S_4$ and (b) $Cu_3Co_6S_8$ at various scanning rates.

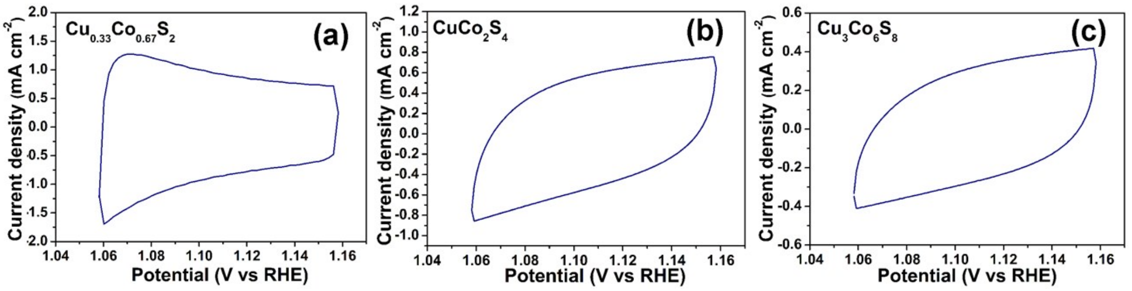

Fig.S7. Cyclic voltammograms of (a) $Cu_{0.33}Co_{0.67}S_2$, (b) $CuCo_2S_4$ and (c) $Cu_3Co_6S_8$ at 20 mV/s in 1 M KOH**.**

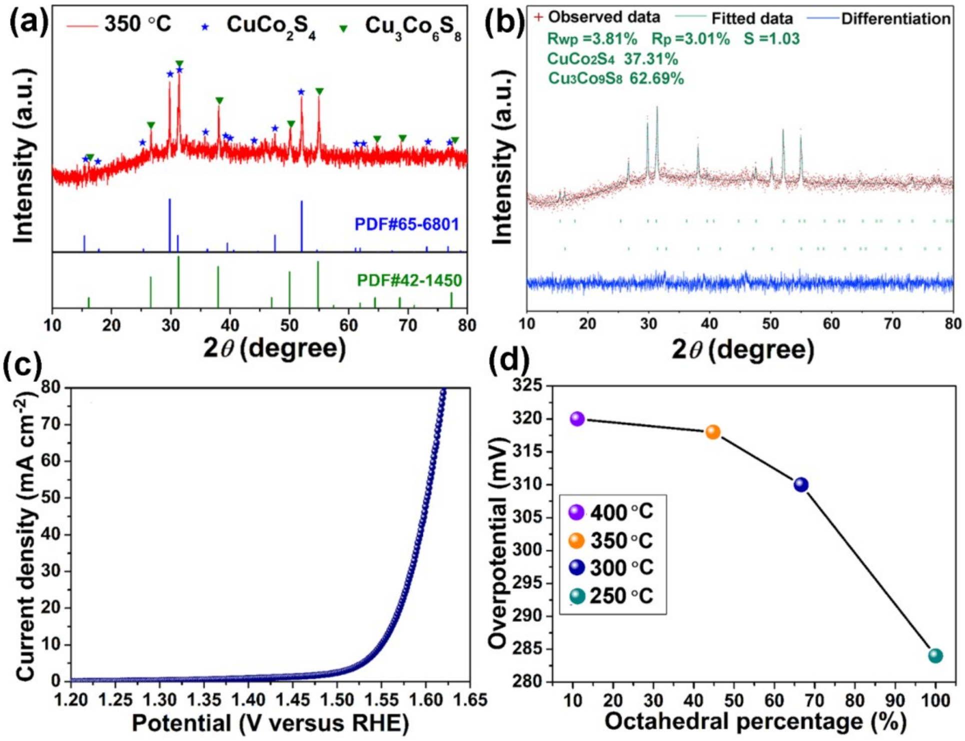

Fig.S8. (a) XRD pattern of the Cu-Co-S product at 350 °C. (b) Rietveld refinement of the XRD pattern of the Cu-Co-S product at 350℃. (c) LSV curve of the product at 350 ℃. (d) The Relationship of the content of octahedron in products at different temperatures with their overpotential performance.

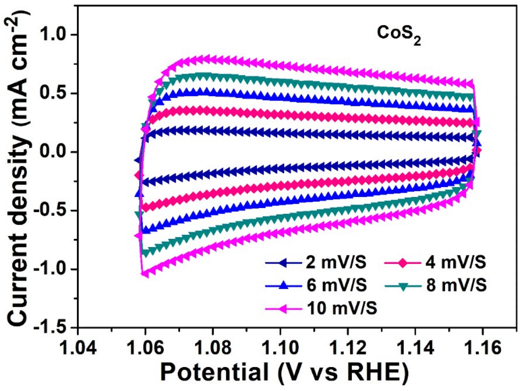

Fig. S9. CVs of CoS_2 at different scan rates.

Table. S1. Summary of the electrochemical activities of $Cu_{0.33}Co_{0.67}S_2$, $CuCo_2S_4$, $Cu_3Co_6S_8$ and CoS_2 for OER.

Catalyst	Overpotential@10 mA cm^{-2} (mV)	Tafel slope (mV dec^{-1})	C_{dl} (mF cm^{-2})	TOF (s^{-1})	R_{ct}
$Cu_{0.33}Co_{0.67}S_2$	284	86	76.32	13.512 s^{-1} @280 mV<br>19.814 s^{-1} @300 mV<br>32.045 s^{-1} @320 mV<br>49.940 s^{-1} @340 mV	47
$CuCo_2S_4$	310	90	31.26	6.701 s^{-1} @280 mV<br>9.799s^{-1} @300 mV<br>16.156s^{-1} @320 mV<br>27.518 s^{-1} @340 mV	54
$Cu_3Co_6S_8$	320	91	15.56	4.244 s^{-1} @280 mV<br>6.548 s^{-1} @300 mV<br>11.099 s^{-1} @320 mV<br>19.570 s^{-1} @340 mV	72
CoS_2	343	98	67.03	2.081 s^{-1} @280 mV<br>2.8112 s^{-1} @300 mV<br>4.905 s^{-1} @320 mV<br>9.638 s^{-1} @340 mV	48

Table. S2. Rietveld refinement results for the XRD patterns of the $Cu_{0.33}Co_{0.67}S_2$ and CoS_2.

Sample	Phase	Space group	Lattice parameters			Amount (wt%)	S
			a(Å)	***b***(Å)	***c***(Å)		
CoS_2	CoS_2	*Pa-3*	5.524	5.524	5.524	100	1.10
$Cu_{0.33}Co_{0.67}S_2$	CoS_2	*Pa-3*	5.638	5.638	5.638	100	1.05

Table. S3. Comparison of OER performances of $Cu_{0.33}Co_{0.67}S_2$ with other reported similar non-noble metal OER electrocatalysts.

Material	Electrolyte (KOH)	Scan rate (mV s^{-1})	η_{10} (mv)	Ref.
$Co_2P@Co_3O_4$	1.0 M	5	335	1
Co_3S_4@NCNTs	0.1 M	5	430	2
CoS_2 NTA/CC	1.0 M	10	276	3
Co-doped Ni–Mn LDH	1.0M	10	310	4
CoS_2 HNSs	1.0 M	10	290	5
$Co_9S_8/Zn_{0.8}Co_{0.2}S@C$	0.1 M	5	292	6
Co9S8@NS-3DrGO	1.0 M	5	317	7
oxygenated-CoS_2–MoS_2	1.0 M	2	272	8
$Co_xS_y@C$	0.1 M	5	470	9
HPMS Co_3O_4/CoS_2	1.0 M	2	280	10
$Zn_xCo_{3-x}O_4$	1.0 M	10	435	11
Co3O4 Nanoflakes	1.0M	5	451	12
$CoSe_2@C$	1.0 M	10	330	13
$CoSe_2$@C-CNT	1.0 M	5	306	14
Zn-Doped $CoSe_2$	1.0 M	2	356	15
CoFeP NSs	1.0 M	5	305	16
octahedral Co_3O_4 particles	1.0 M	2	301	17
Co_3O_4@rGO	1.0 M	2	313	18
CoS_2@N,S-GO	0.1 M	10	390	19
$Cu_{0.33}Co_{0.67}S_2$	1 M	10	284	This work

Reference

1 L. H. Yao, N. Zhang, Y. Wang, Y. M Ni, D. P. Yan and C. W. Hua, *J. Power Sources*, 2018, **374**, 142.

2 H. J. Wang, Z. P. Li, G. H. Li, F. Peng and H. Yu, *CATAL TODAY*, 2015, **245**, 74.

3 C. Guan, X. M. Liu, A. M. Elshahawy, H. Zhang, H. J. Wu, S. J. Pennycook and J. Wang, *Nanoscale Horiz.,* 2017, **2**, 342.

4 Y. Wang, X. H. Liu, N. Zhang, G. Z. Qiu and R. Z. Ma, *APPL CLAY SCI*, 2018, **165**, 277.

5 X. Y. Ma, W. Zhang, Y. D. Deng, C. Zhong, W. B. Hu and X. P. Han, *Nanoscale*, 2018, **10**, 4816.

6 Z. L. Chen, M. Liu, R. B. Wua, *J. CATAL*, 2018, **361**, 322.

7 Y. Li, Y. Z. Zhou, H. J. Wen, J. Yang, C. Maouche, Q. Q. Liu, Y. Y. Wu, C. C. Cheng, J. Zhu and X. N. Cheng, *DALTON T.*, 2018, **47**, 14992.

8 J. G. Hou, B. M. Zhang, Z. W. Li, S. Y. Cao, Y. Q. Sun, Y. Z. Wu, Z. M. Gao and L. C. Sun, *ACS CATAL*, 2018, **8**, 4612.

9 B. L. Chen, R. Li, G. P. Ma, X. L. Gou, Y. Q. Zhu and Y. D. Xia, *Nanoscale*, 2015, **7**, 20674.

10 M. M. Guo, K. Xu, Y. H. Qu, F. Y. Zeng and C. L. Yuan, *ELECTROCHIM ACTA*, 2018, **268**, 10.

11 L. Wang, T. J. Meng, C. X. Chen, Y. W. Fan, Q. Q. Zhang, H. Wang and Y. F. Zhang, *J COLLOID INTERF SCI,* 2018, **532**, 650.

12 S. Q. Chen, Y. F. Zhao, B. Sun, Z. M. Ao, X. Q. Xie, Y. Y. Wei and G. X.Wang, *ACS APPL MATER INTER*, 2015, **7**, 3306.

13 X. B. Liu, Y. C. Liu and L. Z. Fan, *J. Mater. Chem. A.*, 2017, **5**, 15310.

14 M. Y. Yuan, M. Wang, P. L. Lu, Y. Sun, S. Dipazir, J. X. Zhang, S.W. Li and G. J. Zhang, *J COLLOID INTERF SCI*, 2019, **533**, 503.

15 Q. C. Dong, Q. Wang, Z. Y. Dai, H. J. Qiu and X. C. Dong, *ACS APPL MATER INTER*, 2016, **8**, 26902.

16 H. Xua, J. J. Wei, C. F. Liu, Y. P. Zhang, L. Tian, C. q. Wang and Y. k. Du, *ELECTROCHIM ACTA*, 2018, **288**, 82.

17 K. Wu, D. Z. Shen, Q. T. Meng and J. H. Wang, *J COLLOID INTERF SCI*, 2018, **530**, 146.

18 Y. F. Zhao, S. Q. Chen, B. Sun, D.W. Su, X. D. Huang, H. Liu, Y. M. Yan, K. N. Sun and G. X. Wang, *SCI REP*, 2015, **5**, 7629.

19 P. Ganesan, M. Prabu, J. Sanetuntikul and S. Shanmugam, *ACS CATAL*, 2015, **5**, 3625
